# Towards Upscaling of La_5.5_WO_11.25−δ_ Manufacture for Plasma Spraying-Thin Film Coated Hydrogen Permeable Membranes

**DOI:** 10.3390/membranes10090192

**Published:** 2020-08-19

**Authors:** Sonia Escolástico, Cecilia Solís, Antonio Comite, Fiorenza Azzurri, Malko Gindrat, Stefan Moser, Johannes Rauch, Gregory Szyndelman, Rajiv Damani, Jose M. Serra

**Affiliations:** 1 Instituto de Tecnología Química, Universitat Politècnica de València-Consejo Superior de Investigaciones Científicas, Avda. Los Naranjos s/n, E-46022 Valencia, Spain; 2 Dipartimento di Chimica e Chimica Industriale, Università degli Studi di Genova, 16146 Genoa, Italy; antonio.comite@unige.it (A.C.); azzurrif@gmail.com (F.A.); 3 Oerlikon Surface Solutions AG, 8808 Pfäffikon, Switzerland; Malko.Gindrat@oerlikon.com (M.G.); Stefan.Moser@oerlikon.com (S.M.); 4 Oerlikon Metco WOKA GmbH, 36456 Barchfeld, Germany; Johannes.Rauch@oerlikon.com; 5 Oerlikon Metco AG (Switzerland), 5610 Wohlen, Switzerland; Gregory.Szyndelman@oerlikon.com; 6 Sulzer Markets and Technology Ltd., 8404 Winterthur, Switzerland; damani@bluewin.ch

**Keywords:** lanthanum tungstate, H_2_ separation, ceramic protonic conductors, membrane reactors, low-pressure plasma spraying (LPPS-TF)

## Abstract

Lanthanum tungstate (La_6_WO_12_) is a promising material for the development of hydrogen separation membranes, proton ceramic electrolyzer cells and protonic ceramic fuel cells due to its interesting transport properties and stability under different operation conditions. In order to improve the hydrogen transport through the La_6_WO_12_ membranes, thin membranes should be manufactured. This work is based on the industrial production of La_5.5_WO_11.25−δ_ (LWO) powder by spray drying and the manufacturing of thin membranes by low-pressure plasma spraying (LPPS-TF) technique. LPPS-TF allows the production of dense thin films of high quality in an industrial scale. The powders produced by spray drying were morphological and electrochemically characterized. Hydrogen permeation fluxes of a membrane manufactured with these powders were evaluated and fluxes are similar to those reported previously for LWO powder produced in the lab scale. Finally, the transport properties of LWO thin films deposited on Al_2_O_3_ indicate that LPPS-TF produces high-quality LWO films with potential for integration in different applications.

## 1. Introduction

In the last years, protonic conductors (pure and mixed protonic-electronic) have focused the attention of different scientific groups due to their interesting application as hydrogen-selective membranes, electrolysers, fuel cells and catalytic membrane reactors at high temperatures (>400 °C) [1,2,3,4]. The selection of the material for hydrogen permeable membranes based on oxides follows the next criteria: (1) hydrogen permeability, which is related to the ambipolar conductivity of the oxide; (2) stability in harsh gas environments, i.e., reducing environments containing high CO_2_ concentration and several ppm H_2_S; (3) flexibility in the oxide stoichiometry in order to tolerate compositional deviations during the powder manufacture and layer deposition; (4) purity and cost of raw materials; and (5) novelty and intellectual property (IP) opportunities.

Lanthanide tungstates (Ln_6_WO_12_) partly fulfil these requirements, i.e., (1) they present remarkable H_2_ flows and infinite permselectivity [5,6,7], (2) the stability in CO_2_ containing atmospheres and under H_2_S at ppm level [8,9,10] has been demonstrated and (3) the molecular formula presents some flexibility in the ratio Ln/W [11,12] which could favour the flexibility of the industrial production.

Amongst the different lanthanide tungstates, La_5.5_WO_11.25−δ_ has been employed as hydrogen permeable membrane [8] and as electrolyte for protonic fuel cells [13,14]. In addition, this compound has been employed in the fabrication of mixed protonic electronic composites made of La_5.5_WO_11.25−δ_ La_0.87_Sr_0.13_CrO_3−δ_ (LWO-LSC) that present one of the highest H_2_ fluxes reported up to now [15,16,17].

In order to improve the hydrogen flux through the La_5.5_WO_11.25−δ_ membranes, thin membranes should be manufactured. Dense and thin La_5.5_WO_11.25−δ_ based membranes have been obtained by different manufacturing process such as tape casting [18,19] or atomic layer deposition [20].

Another interesting technology for layer deposition is low pressure plasma spraying-thin film (LPPS-TF) that operates at low pressure (around 1–2 mbar) allowing high-throughput and homogeneous coating of large areas (up to 1 m^2^) [21].

This study is based on the industrial production of La_5.5_WO_11.25−δ_ (called in the next LWO) material by spray drying and LWO thin membranes by LPPS-TF technique. LWO powder production on a prototype plant was performed and the obtained powders were morphological and electrochemically characterized. In addition, LWO bulk membranes were manufactured using the powder obtained by spray drying. The reached hydrogen permeation fluxes are similar to those previously reported for LWO materials produced at the lab scale. Finally, LWO thin films were deposited on dense Al_2_O_3_ substrates and electrochemically characterized.

## 2. Materials and Methods

### 2.1. Synthesis

**Figure 1:**
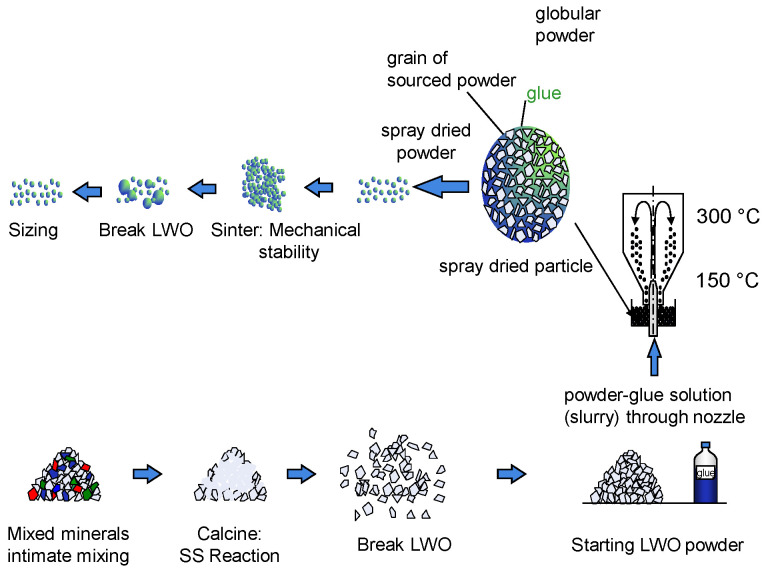
Description of the manufacturing process of the LWO powder.

The precursor materials, lanthanum oxide and tungsten oxide, were intimately mixed in the appropriate stoichiometric proportions and then heat treated to form the target composition and phase. The resulting mixture was broken up and spray dried to form agglomerates. Subsequently, the agglomerates were sintered at 1400 °C for 24 h with the purpose of increase mechanical strength. Finally, the sintered agglomerates were broken up and sized to produce powders of suitable size for thermal spraying. Figure 1 shows a scheme of the manufacturing process.

Three fractions with different mean diameter were obtained after sizing using a binder (glue). The nomenclature and the mean diameter of these three fractions are described in Table 1.

### 2.2. Powder Characterization

Morphological analysis, particle size distributions and elemental semi-quantitative analyses of the obtained powder fractions were carried out by means of a field emission scanning electron microscope (FE-SEM, Supra 40 VP, Carl ZeissAG, Oberkochen, Germany) equipped with both a back scattering detector (Centaurus BSE detector, Deben UK Ltd., Bury St. Edmunds, UK) and an EDS system (cooled Si/Li detector, INCA Energy 450, Oxford Instruments Nanoanalysis, High Wycombe, UK).

Transmission electron microscopy (TEM, JEM 2010, JEOL Ltd., Tokyo, Japan) equipped with an EDS probe (Oxford Link Pentafet Si/Li detector, Abingdon, UK) was used for sample characterization at a nanoscale level. The sample was grinded through an agate mortar, then was dispersed in isopropanol and subsequently was transferred onto a lacey carbon copper grid.

Specific surface area was determined by N_2_ physisorption at 77 K (ASAP 2010, Micromeritics Instrument Corporation, Norcross, Atlanta, GA, USA). The density was obtained by the pycnometer method and the point of zero charge (PZC) was determined by the potentiometric mass titration method [22].

Crystalline phase of the samples was characterized by X-ray diffraction (XRD). The measurements were carried out by an X’Pert PRO diffractometer (PANalytical, Malvern, UK) using CuKα1,2 radiation and an X’Celerator detector in Bragg-Brentano geometry. The XRD patterns were analyzed using X’Pert Highscore Plus software.

Total conductivity was measured on sintered bars by standard four-point DC technique. Rectangular bars used in conductivity measurements were accomplished using the LWO materials uniaxially pressed at 100 MPa and subsequently sintered at 1500 °C, reaching a density of around 99%. Silver paste and wires were used for contacting. The constant current was supplied by a programmable current source (2601, Keithley, Cleveland, OH, USA) while the voltage drop was detected by a multimeter (Keithley 3706).

Total conductivity was measured in reducing and oxidizing atmospheres and the hydration and the isotopic effect was studied. The employed atmospheres were: (1) dry and wet 5% H_2_ in Ar and 5% D_2_ in Ar (where wet means 2.5% H_2_O and 2.5% D_2_O, respectively) and (2) dry O_2_ and O_2_ saturated with H_2_O and D_2_O (2.5%).

### 2.3. Hydrogen Permeation in Bulk Membranes

A gastight membrane was manufactured by using the LWO-2 powder fraction. Powder was pressed at 72 MPa and the green pellet obtained was sintered at 1550 °C for 6 h. Thickness of the LWO membrane was 900 μm and the diameter 15 mm. Gastightness of the membrane was confirmed by helium leakage test and the membrane was totally dense. In order to improve the catalytic activity of the surface, both membrane sides were screen-printed with a 20 μm layer of a Pt ink (Mateck, Jülich, Germany).

Permeation measurements were performed on a double chamber quartz reactor [5,6,7]. 150 mL·min^−1^ of Ar and 100 mL·min^−1^ of a mixture of H_2_-He were employed as sweep and feed gas, respectively. Feed and sweep were humidified by saturation at 20 °C. The hydrogen and helium content in the permeate side was analyzed using a CP-4900 micro-GC (Varian, Santa Clara, CA, USA) equipped with Molsieve5A (Varian, Santa Clara, CA, USA) and Pora Plot-Q (Varian, Santa Clara, CA, USA) glass capillary modules. Despite mixed protonic-electronic conducting based membranes present a theoretical infinite perm-selectivity, some leaks can occur through the sealing. Then, H_2_ permeation was calculated by subtracting the detected He to the total H_2_ flow (H_2_/He ratio varies from 4 to 25 depending on the membrane, temperature and concentration in the feed). Sealing was accomplished by using gold gaskets. Membrane was heated up to 1060 °C and a spring load was applied on the membrane.

### 2.4. Development and Electrochemical Characterization of Thin Films

Thin films were deposited by using the LPPS-TF prototype located at Sulzer Metco in Switzerland. The prototype is composed of a vacuum chamber, a plasma torch O3CP and a flexible 4-axis plasma gun manipulator combined with a horizontally moving sting axis and equipped with high speed CCD camera, IR-pyrometer, IR-camera and high end optical spectrometer. More details of the prototype can be found elsewhere [23].

Three different LWO films were deposited on Al_2_O_3_ substrates by LPPS-TF. Under the same deposition conditions, denser films are obtained by using smaller particles [24]. Therefore, LWO-1 fraction that possesses lower particle size was employed for the deposition experiments. The parameters employed in the deposition and the mean thickness of the resulting films are listed in Table 2. The three depositions were carried out at low chamber pressure of 1.5 mbar (150 Pa). Ar flow, standoff (spray distance) and coating time were modified in the different depositions. Increase of Ar has usually the effect to produce denser coating as the Ar flow increases also the velocity of molten particles in the plasma jet. The increase of the spray distance allows reducing the temperature and temperature strain on the substrate.

The crystalline phase of the films was analyzed by XRD measurements and total conductivity measurements were performed by using different atmospheres: dry and wet He (saturated at room temperature with H_2_O and D_2_O) and dry and wet 5% of H_2_ and D_2_ in He (saturated with H_2_O and D_2_O, respectively). Morphology of the LWO films was analyzed using field emission scanning electron microscopy (FESEM, Zeiss Ultra 55 Carl Zeiss AG, Germany).

## 3. Results

### 3.1. Powder Characterization

**Figure 2:**
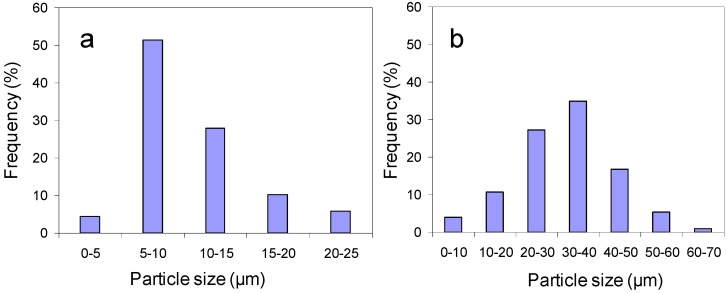
Particle size distribution obtained by SEM image analysis of LWO-1 (**a**) and LWO-2 (**b**).

During the manufacturing process, the obtained powders were sized and three fractions with different mean diameter (see Table 1) were obtained. Figure 2 shows the particle size distributions for LWO-1 and LWO-2 fractions.

About 50% of particles of LWO-1 powder have a diameter between 5 and 10 µm whereas the mean diameter of the LWO-2 particles is mainly comprised between 30 µm and 40µm. LWO-3 fraction is mainly constituted by agglomerates as can be observed in Figure 2 and subsequently the corresponding main diameter particle was not measured.

**Figure 3:**
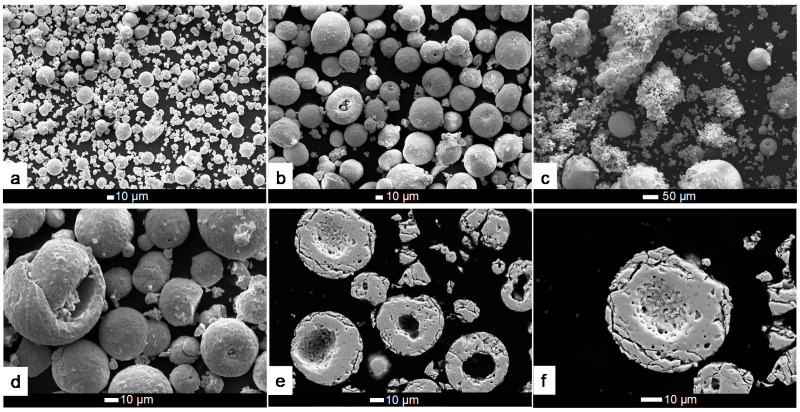
SEM micrographs of the powders named LWO-1 (**a**), LWO-2 (**b**,**d**), LWO-3 (**c**) and the cross sections of LWO-2 (**e**) and LWO-3 (**f**).

Morphology of powders and their cross-section was studied by FE-SEM. The LWO-1 and LWO-2 powders (Figure 3a,b, respectively) are composed of discrete particles, which exhibit a remarkably different size. Figure 3d gives a better idea on the morphological characteristics of the LWO powders, which are spherical in shape and exhibit numerous large pores on their outer surface. Moreover, the LWO particles often reveal an inner cavity as it can be appreciated from the cross sections of the particles shown in Figure 3e,f (the last micrograph with higher magnification). More FE-SEM images can be found in Supporting Information Figures (S1–S3).

**Figure 4:**
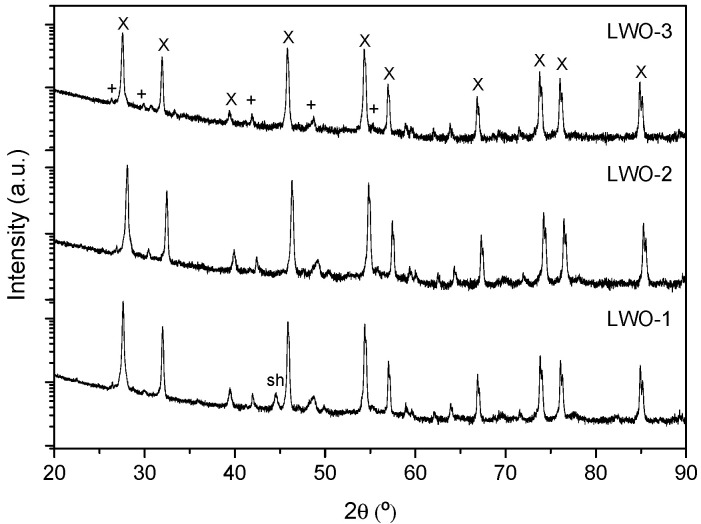
XRD patterns of the three different powder fractions. Note the presence of a diffraction peak around 45° due to the sample holder (sh).

Crystalline phase of the three different fractions was studied by XRD. The corresponding XRD patterns (intensity in log scale in Figure 4) show that the major crystalline phase is the targeted cubic fluorite structure (La_6_WO_12_, X symbols in the figure) while the secondary phase La_10_W_2_O_21_ (+symbol) is present in significant amount. The principal diffraction peaks of both phases are very similar and therefore both structures do not differ substantially. The La_10_W_2_O_21_ presents a large amount of minor diffraction peaks, which suggest the presence of slight symmetry distortion. It is suggested the presence of an orthorhombic superstructure. Additionally, minor amounts of La_2_O_3_ oxide are detected, which is prone to hydrate and could provoke the mechanical failure, if it is still present in the final ceramic component. All three fractions show similar XRD patterns.

In addition, EDS microanalysis was carried out in several points and areas with the aim of gathering information on the stoichiometry of the powders. Table 3 reports the average EDS composition of the three powders. The average stoichiometry seems to be very close to the theoretical formula La_5.5_WO_11.25−δ_. The EDS data were analyzed by keeping into account the eventual presence of other phases with a close La/W ratio (La_6_W_2_O_15_, La_10_W_2_O_21_), and the presence of La_10_W_2_O_21_ phase seems to be possible as already found by the XRD characterization. Only few isolated fragments of rich La phases were found.

**Figure 5:**
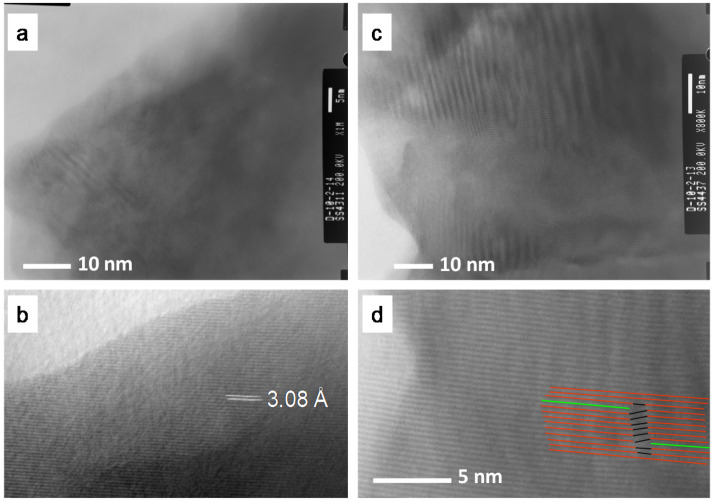
TEM micrographs showing a crystallite of La_2_O_3_ (**a**) along with an evaluation of the interplanar distance (**b**); and a crystallite where La/W = 5.3 (**c**) along with an enlargement where some dislocations are marked (**d**).

The presence of some phase segregations associated to La_2_O_3_ was confirmed by TEM (Figure 5a). The interplanar spacing (Figure 5b) is in good agreement with the literature [25]. Figure 5c shows a crystallite where the ratio La/W is about 5.3 and some dislocations can be found (Figure 5d).

**Figure 6:**
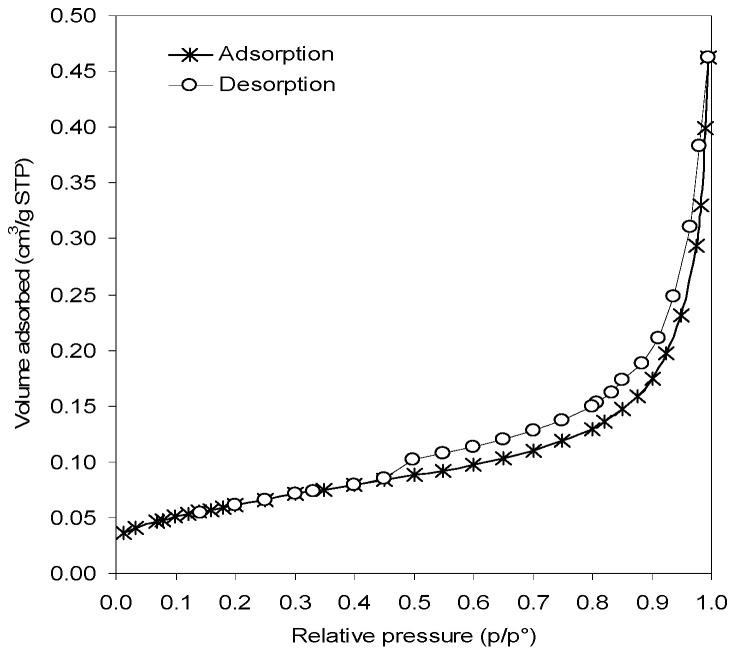
N_2_ adsorption and desorption isotherms for powder LWO-2.

The shape of the adsorption isotherm (Figure 6) reveals the macroporous nature of the spray-dried LWO powders. On the other hand, the desorption gives a hysteresis (type H3) that is due to the presence of aggregations which may generate slit-shaped mesopores with not uniform size and shape. The BET surface area value, as derived by physisorption analyses, is 0.22 m^2^/g.

The point of zero charge (PZC) was determined by using the potentiometric mass titration method. The PZC value of the powder was ~10, comparable to the isoelectric point of La_2_O_3_ (about 10.4 [26]) while is completely different from that of WO_3_ (about 0.2–0.5). The density of the three powders is about 6.29 g/cm^3^ which is in close agreement with the described theoretical density for La_6_WO_12_ [11].

Conductivity measurements were performed in reducing atmospheres in order to validate the appropriate electrochemical properties of the three powder fractions despite the different stoichiometry that present.

**Figure 7:**
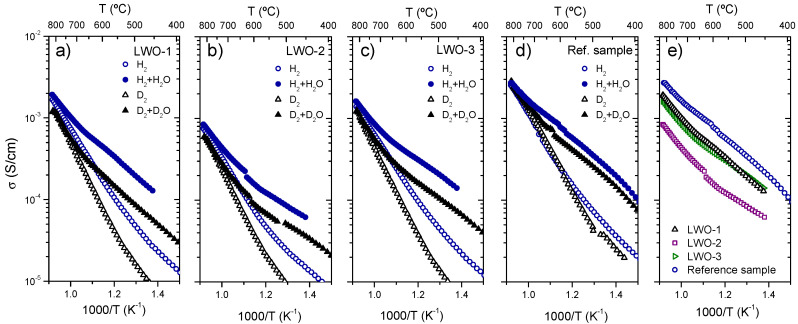
Total conductivity in reducing conditions (H_2_, D_2_, H_2_ + H_2_O and D_2_ + D_2_O where H_2_ and D_2_ are diluted in 95% He) as a function of temperature for the three fractions of LWO (**a**–**c**) and the reference sample (**d**) and conductivity comparison in wet 5%H_2_ in He for the four samples (**e**).

Figure 7 presents the conductivity results obtained for the three fractions and the reference sample (La_5.5_WO_11.25−δ_ synthesized by modified Pechini [8]) in both 5% H_2_ and 5% D_2_ diluted in He (dry and saturated with either H_2_O or D_2_O). 

Protonic transport prevails at temperatures below 700 °C as can be ascertained from the hydration effect ($\sigma_{H_{2}+H_{2}O}>\sigma_{H_{2}}$ and $\sigma_{D_{2}+D_{2}O}>\sigma_{D_{2}}$) and the H/D isotopic effect ($\sigma_{H_{2}+H_{2}O}>\sigma_{D_{2}+D_{2}O}$) observed. On the other hand, in agreement with the reference sample, these effects (hydration and isotopic effect) become negligible at temperatures above 700 °C and an increase of the activation energy is also observed indicating a predominant oxygen ion and n-type electronic transport with respect to proton conduction due to the exothermic dehydration of the sample.

Despite the similar thermal behavior of the three fractions as compared with the reference sample, the total conductivity obtained is lower than in the reference sample as it can be observed in Figure 7e where total conductivity in wet 5% H_2_ diluted in He as a function of the reciprocal temperature is plotted for the four LWO compounds. This lower conductivity can be related to the different La/W stoichiometric ratio of the compounds. In fact, the lower conductivity was obtained for LWO-2 in agreement with Magrasó et al. who reported that conductivity drops in reducing atmospheres when La/W ≤ 5.2 [11]. This decrease in the conductivity values could be related to a lower magnitude of the n-type conductivity because the isotopic effect seems to possess higher magnitude in the three studied fractions win respect to the reference sample.

Conductivity of the samples was also measured under oxidizing conditions. Figure S4 shows the conductivity results obtained for the three different fractions in oxygen (dry and saturated with either H_2_O or D_2_O). The three fractions show the clear effect of the oxide hydration and the H/D isotopic effect, especially important at temperatures below 750 °C. In general, these three samples show similar conduction behavior and activation energy. Total conductivity values of LWO-1 and LWO-2 samples are very similar to those corresponding to the reference sample.

From the conductivity measurements, it can be concluded that the powder samples produced by spray drying for the subsequent manufacture of membranes are good proton conductors and present certain electronic conductivity in reducing atmospheres. In principle, it is expected that their ambipolar conductivity should be adequate for hydrogen separation at high temperatures, at least as adequate as the reference LWO. With the aim of confirm this last statement, hydrogen permeation measurements were performed by using a membrane made of LWO-2 powder which presents the lower conductivity values obtained.

### 3.2. Hydrogen Permeation Test

**Figure 8:**
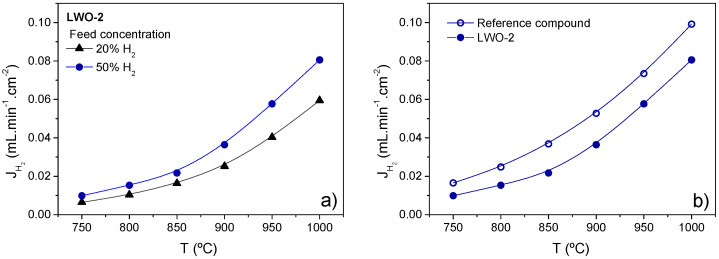
H_2_ permeation as a function of temperature for LWO-2 feeding different H_2_ concentrations (**a**) and LWO-2 and reference membranes feeding 50% H_2_ (**b**). Lines are guide for reader’s eye.

Figure 8a illustrates the temperature dependence of hydrogen permeation at different *p*H_2_ when both sides of the membrane are humidified at room temperature using a membrane made of LWO-2 powder. H_2_ flow increases with increasing *p*H_2_ in the feed stream as it is expected from Wagner equation. On the other hand, hydrogen permeation flows are slightly lower than those obtained with the reference sample [8] as shown in Figure 8b. The reference sample powder was synthesized by the Pechini modified method and the membrane was obtained by pressing the powder at 72 MPa and sintering the green pellet at 1550 °C for 6 h. Due to the important H_2_ flows reached, LWO based membranes are presented as promising hydrogen permeable membranes if thin supported membranes could be manufactured with these compounds.

### 3.3. LWO Thin Films Characterization

In order to study the viability as ionic membranes of the supported LWO thin films made by LPPS-TF, three different LWO films were deposited on Al_2_O_3_ substrates by using different parameters in the deposition process (Table 2).

**Figure 9:**
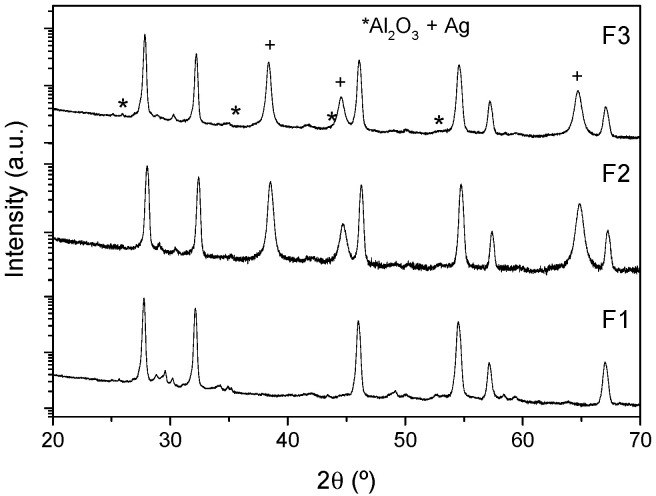
XRD patterns of F1, F2 and F3 LWO films deposited on Al_2_O_3_.

The structural characterization of the different LWO films deposited by LPPS-TF on Al_2_O_3_ substrates was performed by XRD. Figure 9 shows XRD patterns (intensity in log scale) corresponding to F1, F2 and F3 LWO films. The films show the peaks corresponding to the LWO phase together with some of the Al_2_O_3_ substrate and the Ag metallic contacts used for conductivity measurements in the case of the F2 and F3 samples (labeled with an * and +respectively). Some differences are observed among these different films, mainly some small peaks that appear in the range of 28–31°. These peaks, principally observed in the F1 sample, can be assigned to a small amount of La_6_W_2_O_15_ phase. Otherwise, F3 sample does not present so many peaks in this range, and only has a small peak at 30.3° also observed in the powder samples and that could be attributed to the LWO phase.

**Figure 10:**
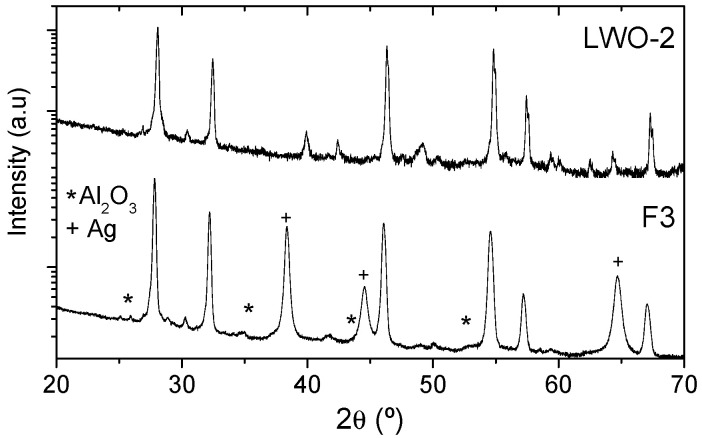
XRD patterns of powder LWO-2 and F3 LWO film.

If XRD patterns of the films are compared with those of the powders (Figure 4) it can be ascertained that the XRD pattern of sample F3 is very similar to that of the powder LWO-2 as can be directly compared in Figure 10, where only these two samples are represented. F3 LWO film does not present impurities and the cell parameters are a bit longer, but very close, than those of the powder of sample LWO-2 (which has the shortest cell parameter of the measured powders) as can be concluded for the position of the diffraction peaks.

**Figure 11:**
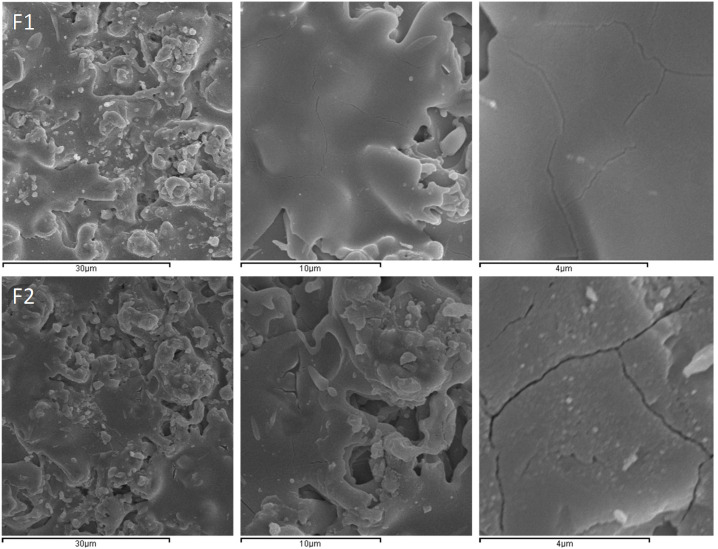
SEM images of the F1 (**top**) and F2 (**bottom**) films.

SEM images of the F1 and F2 films are shown in Figure 11, top and bottom, respectively. The lowest magnification images correspond to dense layers but the highest magnification images show small cracks in the layers. These cracks are not found along the whole surface of the samples but sometimes look quite deep (as in the case of the F2 sample) and may be associated with the mismatch between the thermal expansion of the film (11.1 × 10^−6^ K^−1^) and the Al_2_O_3_ substrate (7–8 × 10^−6^ K^−1^).

**Figure 12:**
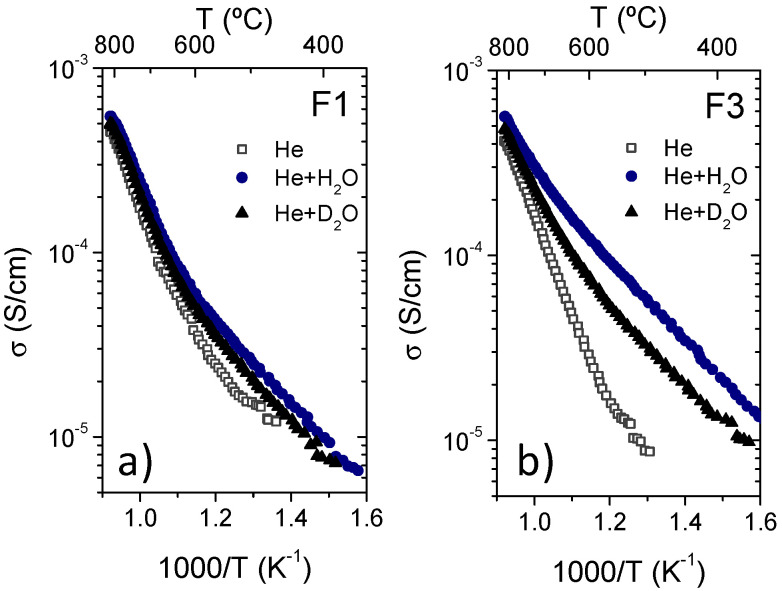
Total conductivity as a function of the temperature of F1 (**a**) and F3 (**b**) LWO films in dry He and in He saturated at room temperature with H_2_O and D_2_O.

**Figure 13:**
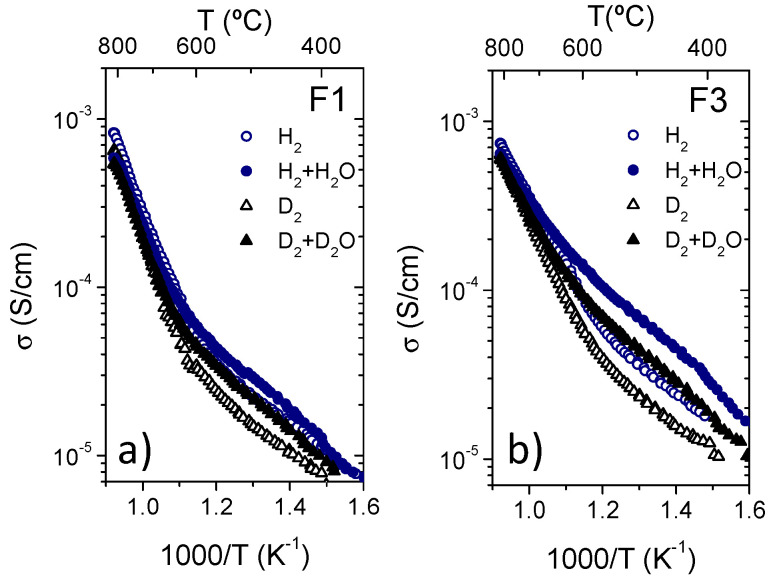
Total conductivity as a function of the temperature of F1 (**a**) and F3 (**b**) LWO films in different dry and wet reducing atmospheres (5% of H_2_ and D_2_ in He with H_2_O and D_2_O respectively when necessary).

In order to complete the films characterization, the transport properties of the films were studied by total conductivity measurements under different atmospheres. The dependence of the total conductivity (σ) with the inverse of the temperature for F1 and F3 films is presented in Figure 12 under dry and wet He and in Figure 13 under dry and wet H_2_ and D_2_ (were 2.5% of both H_2_O and D_2_O have been used for wet atmospheres when necessary).

As in the case of the pressed samples from the spray-dried powder, in both samples and under the different atmospheres two major trends can be distinguished below 700 °C: (1) the hydration effect (the total conductivity is higher in wet than in dry atmospheres); and (2) a strong isotopic effect (total conductivity in wet atmospheres with H_2_O is higher than with D_2_O). Both effects clearly indicate that the protonic conductivity is predominant in this material under wet conditions at temperatures below 700 °C, regardless of the oxygen partial pressure, as reported elsewhere [8,27]. However the isotopic effect becomes almost negligible at higher temperatures and there is an increase of the activation energy at around 600 °C as can be inferred from the change in the slope of the conductivity at this temperature (Figure 12 and Figure 13). According to reported data of the partial conductivities [27] this change in the *E_a_* can be ascribed to a change of the predominant charge carriers (protonic, oxygen-ionic and electronic conductivities). Activation energies (*E_a_*) and pre-exponential factor (*A*) of the F1 and F3 LWO films measured under different atmospheres are summarized in Table 4. The increase of the *E_a_* above 600 °C is ascribed to both electronic (p-type in He and n-type in H_2_) and oxygen-ion conduction, which becomes predominant due to the oxide dehydration at high temperatures.

On the other hand, samples F1 and F3 show some differences. The total conductivity of sample F3 is always higher and presents stronger hydration and isotopic effects than the F1 sample. This small conductivity disagreement (smaller than a factor 1.5) can only be assigned to the small differences observed in the crystalline structure analysis. In fact, the F1 film has some impurities of the La_6_W_2_O_15_ phase, which presents lower conductivity values [28] than those of LWO phase, explaining these differences.

**Figure 14:**
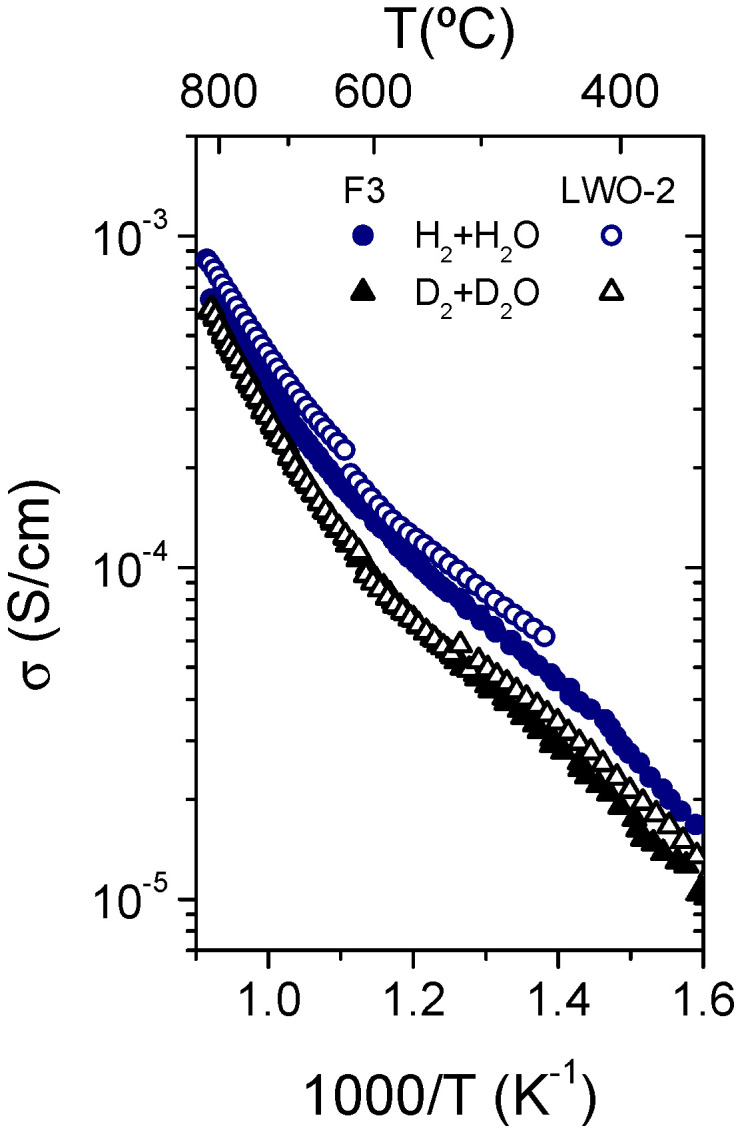
Comparison of the total conductivity as a function of the temperature of the F3 LWO film and the powder LWO-2 measured in H_2_ + H_2_O and D_2_ + D_2_O.

As F3 LWO film has the same structure as the sample LWO-2, Figure 14 compares the total conductivities of both samples measured in H_2_ + H_2_O and D_2_ + D_2_O, which coincide in the whole range of temperature. This fact confirms the technical viability of using LPPS-TF for manufacturing of supported LWO membranes in industrial applications.

## 4. Conclusions

Spray-dried LWO powders used for thin film deposition by LPPS-TF were manufactured in a prototype plant to ensure that any developed material can be successfully up-scaled for industrial production. The obtained powders meet specification requirements for LPPS-TF processing in terms of average particle size and particle size distribution, and show sufficient phase purity and good ambipolar conductivity. Bulk membranes were prepared by using these materials and the reached hydrogen permeation flows were very similar to those corresponding to LWO lab scale manufacture. The viability of the LWO thin supported membranes produced by the LPPS-TF was evaluated. These films show the same transport properties, confirming the quality of the films growth with this technique despite the presence of cracks observed by SEM. By using supported thin film membranes manufactured by LPPS-TF, H_2_ flows around 1.014 mL·min^−1^·cm^−2^ at 1000 °C (thickness of 71 µm) are expected. From the obtained results, it can be inferred that LPPS-TF produces highly hydrogen-permeable films that can allow the application of LWO membranes as catalytic membrane reactors in different industrial processes.

## Figures and Tables

**Table 1:** Nomenclature and mean diameter of the three powder fractions obtained in the manufacture process.

Sample ID	Mean Diameter (µm)
LWO-1	<30
LWO-2	>30
LWO-3	>45

**Table 2:** Parameters employed in the deposition of three different LWO films on Al_2_O_3_ substrates by LPPS-TF.

Lwo Films		Plasma Parameter					Powder		Other		
Run	Thickness (µm)	Ar Flow (L/min)	He Flow (L/min)	H_2_ Flow (L/min)	Current (A)	Power (kW)	Feed Rate (g/min)	Carrier Gas (L/min)	Standoff (mm)	O_2_ Flow (L/min)	Coating Time (s)
F1	77	100	20	0	2600	118	2 × 20	2 × 6	1000	2	45
F2	120	120	20	0	2600	123	2 × 20	2 × 6	1000	2	60
F3	71	120	20	0	2600	123	2 × 20	2 × 6	1300	2	60

**Table 3:** Atomic percentage and stoichiometric composition from EDS analyses.

At % Element	LWO-1	LWO-2	LWO-3
La	30.8	30.5	31.4
W	5.7	5.9	5.4
O	63.5	63.6	63.2
Formula	La_5.3_WO_11_	La_5.1_WO_10.8_	La_5.8_WO_11.7_

**Table 4:** *E_a_* and *A* of the F1 and F3 LWO films.

Atmosphere	F1				F3			
	350–600 °C		600–800 °C		350–600 °C		600–800 °C	
	*A*	*E_a_*	*A*	*E_a_*	*A*	*E_a_*	*A*	*E_a_*
	(S·K/cm)	(eV)	(S·K/cm)	(eV)	(S·K/cm)	(eV)	(S·K/cm)	(eV)
He	190	0.66	183,000	1.19	21,800	1.03	71,000	1.12
He + H_2_O	91	0.56	22,000	0.98	79	0.50	1700	0.74
He + D_2_O	43.8	0.52	92,000	1.12	53	0.51	5400	0.86
H_2_	21	0.47	680,000	1.26	26.8	0.43	15,000	0.89
H_2_ + H_2_O	17	0.44	48,000	1.05	44.5	0.45	2100	0.75
D_2_	16	0.48	1.4 × 10^6^	1.36	25	0.45	74,000	1.05
D_2_ + D_2_O	15	0.45	4.2 × 10^5^	1.25	42	0.45	8400	0.86

## Supplementary Materials

The following are available online at https://www.mdpi.com/2077-0375/10/9/192/s1, Figure S1: SEM micrograph of the powders named LWO-1; Figure S2: SEM micrograph of the powders named LWO-2; Figure S3: SEM micrograph of the powders named LWO-3; Figure S4: Total conductivity in oxidizing conditions (O_2_, O_2_ + H_2_O and O_2_ + D_2_O) as a function of temperature for the three fractions of LWO (a,b,c) and conductivity comparison in wet O_2_ for the three fractions and the reference sample (d).
