# Supplementary material for: Towards Upscaling of La5.5WO11.25−δ Manufacture for Plasma Spraying-Thin Film Coated Hydrogen Permeable Membranes
Source: Membranes (Basel). 2020 Aug 19;10(9):192. doi: 10.3390/membranes10090192 (PMC7560138; doi:10.3390/membranes10090192)
Supplement: Supplementary file 1 [file membranes-10-00192-s001.pdf]

membranes

Supplementary Materials: Towards Upscaling of $La_{5.5}WO_{11.25-\delta}$ Manufacture for Plasma Spraying-Thin Film Coated Hydrogen Permeable Membranes

Sonia Escolástico [1,*], Cecilia Solís [1,*], Antonio Comite [2], Fiorenza Azzurri [2], Malko Gindrat [3], Stefan Moser [3], Johannes Rauch [4], Gregory Szyndelman [5], Rajiv Damani [6] and Jose M. Serra [1,*]

[1] Instituto de Tecnología Química, Universitat Politècnica de València-Consejo Superior de Investigaciones Científicas, Avda. Los Naranjos s/n, E-46022 Valencia, Spain
[2] Dipartimento di Chimica e Chimica Industriale, Università degli Studi di Genova, 16146 Genoa, , Italy; antonio.comite@unige.it (A.C.); azzurrif@gmail.com (F.A.)
[3] Oerlikon Surface Solutions AG, 8808 Pfäffikon, Switzerland; Malko.Gindrat@oerlikon.com (M.G.); Stefan.Moser@oerlikon.com (S.M.)
[4] Oerlikon Metco WOKA GmbH, 36456 Barchfeld, Germany; Johannes.Rauch@oerlikon.com
[5] Oerlikon Metco AG (Switzerland), 5610 Wohlen, Switzerland; Gregory.Szyndelman@oerlikon.com
[6] Sulzer Markets and Technology Ltd., 8404 Winterthur, Switzerland; damani@bluewin.ch
* Correspondence: soesro@itq.upv.es (S.E.); cecilia.solis@frm2.tum.de (C.S.); jmserra@itq.upv.es (J.M.S.)

Received: 7 July 2020; Accepted: 3 August 2020; Published: date

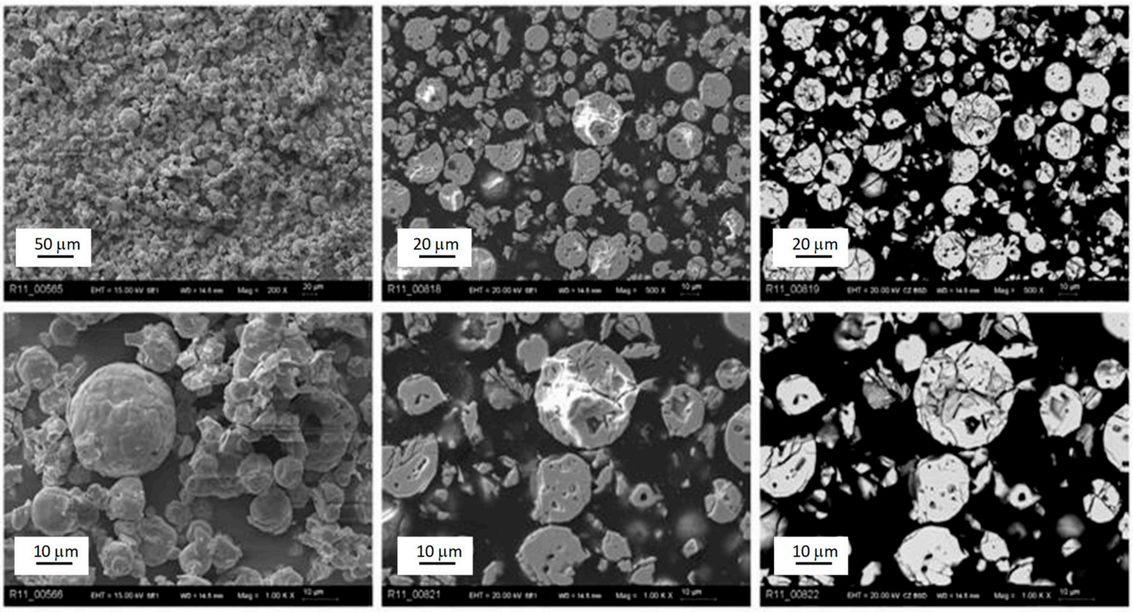

Figure S1. SEM micrograph of the powders named LWO-1.

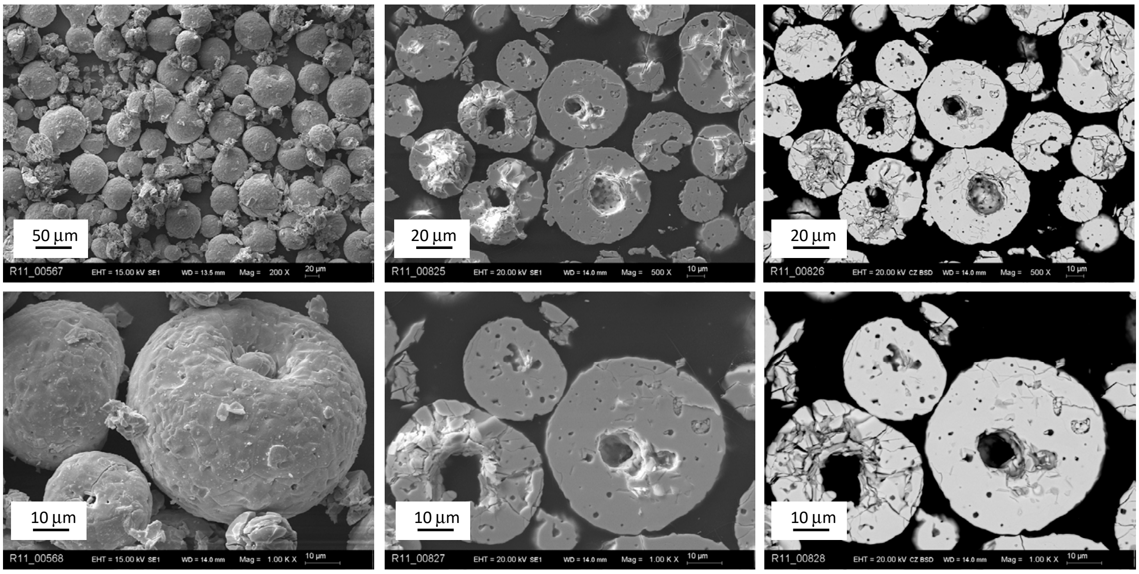

Figure S2. SEM micrograph of the powders named LWO-2.

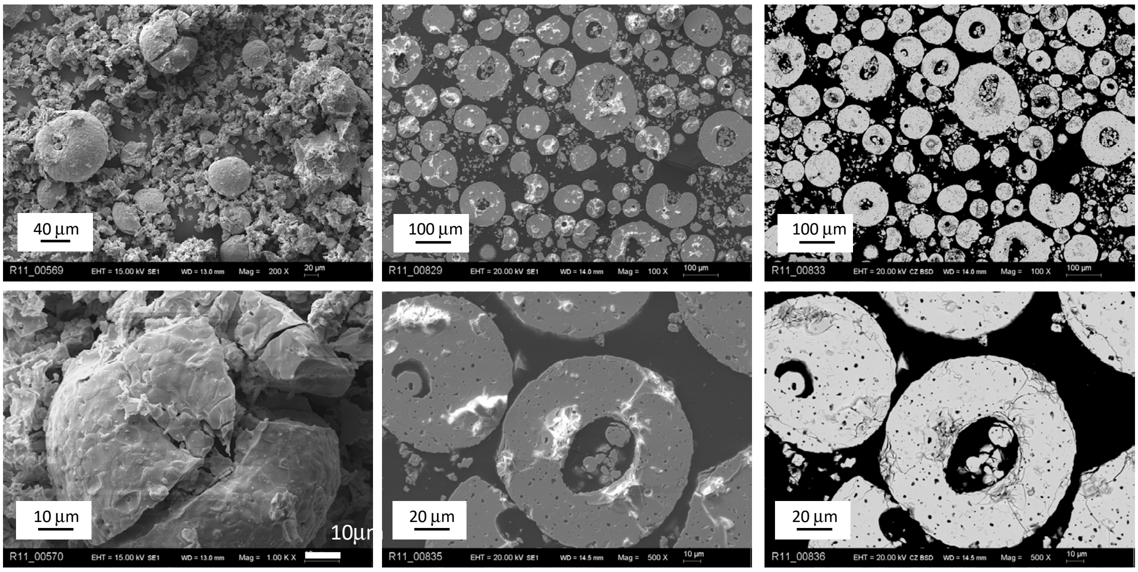

Figure S3. SEM micrograph of the powders named LWO-3.

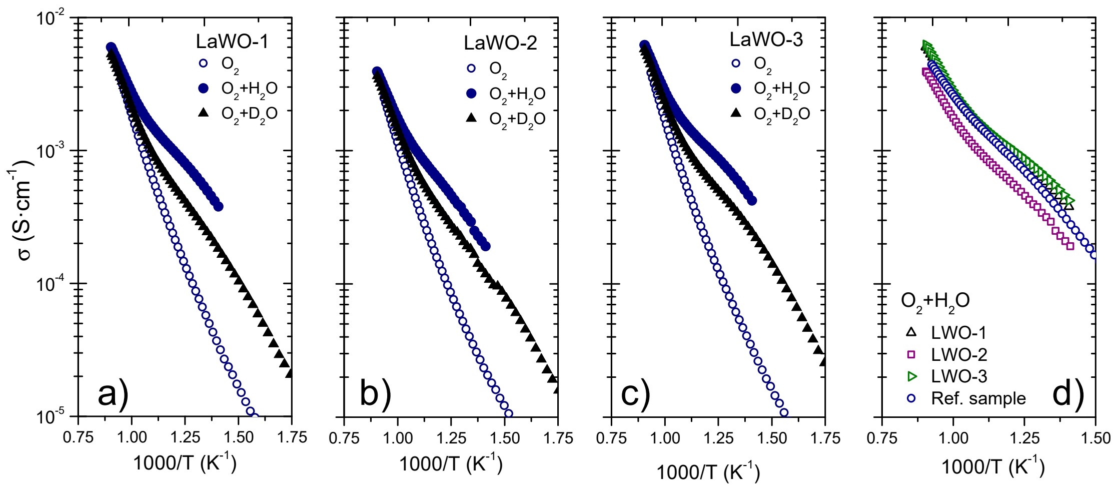

Figure 4. Total conductivity in oxidizing conditions (O_2, O_2+H_2O and O_2+D_2O) as a function of temperature for the three fractions of LWO (**a**,**b**,**c**) and conductivity comparison in wet O_2 for the three fractions and the reference sample (**d**).
